# Spectral flow cytometry for high-dimensional immune profiling: from technological advances to multimodal precision immunology

**DOI:** 10.3389/fimmu.2026.2005073

**Published:** 2026-10-26

**Authors:** Yonglan Zhu, Jiaxuan Li, Chunchun Li, Meirong Yu, Amin Liu, Qin Zhang, Yuqiong Xie, Keda Chen

**Affiliations:** 1 Center for Basic Research, The Second Affiliated Hospital, School of Medicine, Zhejiang University, Hangzhou, China; 2 Key Laboratory of Artificial Organs and Computational Medicine in Zhejiang Province, Shulan International Medical College, Zhejiang Shuren University, Hangzhou, China

**Keywords:** high-dimensional cytometry, immune ecosystem, immune heterogeneity, immunophenotyping, multidimensional immune profiling, precision immunology, spectral flow cytometry, spectral unmixing

## Abstract

Spectral flow cytometry has emerged as a powerful high-dimensional platform for resolving immune heterogeneity and functional states at the single-cell level. By capturing the full emission spectra of fluorophores and applying computational spectral unmixing, it expands the multiparameter profiling capacity of conventional flow cytometry and enables more comprehensive characterization of immune cell phenotypes and functional states. In this review, we summarize the technological evolution of spectral flow cytometry and discuss how spectral acquisition and computational unmixing support multidimensional analysis of immune composition, cellular states, and functional programs. We then examine its applications in immune ecosystem mapping, tumor immunology, immune development and regeneration, vaccination, autoimmune diseases, and translational immune monitoring, with particular emphasis on biomarker discovery, disease stratification, therapeutic response assessment, and precision immunology. We further discuss the integration of spectral flow cytometry with mass cytometry, artificial intelligence, single-cell transcriptomics and proteomics, spatial profiling, imaging technologies, and functional assays. These multimodal approaches provide complementary phenotypic, molecular, spatial, and functional information, creating opportunities for more comprehensive reconstruction of immune ecosystems and deeper interpretation of cellular states. Despite these advances, challenges remain in standardization and cross-platform reproducibility, autofluorescence management, high-dimensional data analysis, and clinical validation. Future development will therefore require not only continued advances in spectral acquisition and computational analysis but also harmonized workflows, robust analytical frameworks, and effective multimodal integration. Overall, spectral flow cytometry is evolving from a high-parameter immunophenotyping technology into a scalable platform for ecosystem-level immune profiling, providing an important bridge between cellular phenotyping, mechanistic discovery, biomarker development, and precision immunology.

## 1 Introduction

Immune systems are characterized by extensive cellular heterogeneity, functional plasticity, and dynamic interactions among diverse cell populations. Understanding how individual immune cells differ in lineage, activation state, differentiation status, and functional programs is therefore essential for elucidating immune regulation, disease pathogenesis, and therapeutic responses. Conventional population-level analyses can obscure rare or functionally distinct cell states, whereas single-cell technologies have enabled increasingly resolved characterization of immune phenotypes and cellular states. Among these approaches, flow cytometry remains one of the most widely used platforms for high-throughput single-cell immune profiling because of its speed, multiparameter capacity, experimental accessibility, and established clinical applications (1).

However, the increasing complexity of immunological questions has exposed limitations in conventional fluorescence flow cytometry. As the number of simultaneously measured markers increases, the spectral overlap among fluorophores becomes increasingly difficult to manage within a discrete channel-based detection framework. Compensation can correct spectral spillover under defined experimental conditions, but highly complex panels require careful optimization of fluorophore brightness, antigen expression, detector configuration, and control design (2). These constraints become particularly relevant when the biological objective shifts from identifying major immune lineages to resolving rare populations, co-expression patterns, and continuous functional states within heterogeneous immune ecosystems.

Spectral flow cytometry has emerged as an important evolution of fluorescence-based flow cytometry by changing how fluorescent information is acquired and computationally resolved. Rather than assigning fluorescence signals primarily to predefined optical channels, spectral flow cytometry captures the emission characteristics across a broader spectral range and uses reference spectra and computational unmixing to resolve overlapping fluorescent signatures (3, 4). This approach does not simply increase the number of detectable parameters; more fundamentally, it shifts fluorescence analysis from discrete channel measurement toward information-rich spectral profiling.

This shift also changes the conceptual framework of experimental design. Instead of treating spectral overlap primarily as a constraint that must be minimized, spectral information can be incorporated into computational models to distinguish overlapping fluorescence signatures. As a result, spectral flow cytometry provides greater flexibility for high-dimensional panel design and enables simultaneous interrogation of lineage, differentiation, activation, and functional states within individual cells (5, 6). The resulting data are particularly relevant to systems-level immunology, where immune phenotypes emerge from the coordinated distribution and interaction of multiple cellular populations rather than from a single molecular marker or cell subset.

Despite the rapid adoption of spectral flow cytometry in immunological research, its broader significance within single-cell and systems immunology remains incompletely synthesized. Existing studies have demonstrated its utility in high-dimensional immune phenotyping, tumor immune profiling, immune development, disease monitoring, and cellular therapy, yet these applications are often discussed separately (7–10). A conceptual framework linking the technological transition from channel-based detection to spectral unmixing with the biological questions that can subsequently be addressed is still needed. Moreover, the relationship between spectral flow cytometry and emerging approaches, including artificial intelligence, single-cell omics, and spatial profiling, requires further consideration.

In this Review, we do not aim to provide a conventional catalog of spectral flow cytometry applications. Instead, we examine spectral flow cytometry as an evolving analytical paradigm in immunology, tracing its transition from channel-based fluorescence detection to spectral acquisition and computational unmixing. We then discuss how this transition expands the ability to resolve immune heterogeneity, decode immune ecosystems, investigate immune development and cell fate, and support translational immune profiling. Finally, we examine its integration with artificial intelligence and complementary single-cell and spatial technologies, and highlight the technical, analytical, and standardization challenges that must be addressed for spectral flow cytometry to contribute fully to systems and translational immunology.

## 2 Technology evolution: from channel-based detection to spectral unmixing

### 2.1 Limitations of conventional flow cytometry

Traditional flow cytometry has relied on discrete optical channels to quantify fluorescence signals from labeled cells. Each fluorophore is typically assigned to a specific detector and filter configuration, making multiparameter analysis dependent on instrument optical design and fluorophore characteristics. As the number of measured markers increases, overlapping emission spectra among fluorophores inevitably introduce signal spillover, requiring compensation procedures to correct spectral interference.

Compensation represents an established mathematical strategy for correcting fluorescence spillover based on reference controls, particularly single-stained samples. However, as panel complexity increases, compensation performance becomes increasingly dependent on reference spectral quality, fluorescence intensity, experimental consistency, and careful panel optimization. Consequently, the expansion from conventional phenotyping toward highly multiplexed characterization of rare populations, co-expression patterns, and functional immune states has become increasingly challenging.

Although conventional flow cytometry remains a cornerstone technology for high-throughput single-cell analysis, the growing demand for multidimensional characterization of cellular states has driven the development of complementary high-dimensional approaches, including spectral flow cytometry, mass cytometry, and single-cell sequencing technologies. These platforms differ in signal acquisition principles, analytical dimensions, throughput, and biological information provided (**Table 1**), and they represent complementary strategies for resolving cellular heterogeneity.

**Table 1:** Comparison of major high-dimensional single-cell technologies for immune profiling.

Technology	Detection principle	Typical number of analytes per cell	Information content	Advantages	Limitations	Typical applications
Conventional flow cytometry	Discrete fluorescence channels + compensation	Approximately 8–20 markers in routine multiparameter applications; higher numbers are achievable with specialized instruments and optimized panels	Protein expression	High throughput, mature workflow	Limited parameters, compensation complexity	Routine immunophenotyping
Spectral flow cytometry	Full emission spectra +computational unmixing	Approximately 30-50+ markers in advanced panels; validated panels with 40 or more colors have been reported	High-dimensional protein profiling	Flexible panels, live-cell analysis	Spectral library, data complexity	Immune profiling, tumor immunology
Mass cytometry	Metal isotope-taggedantibodies + TOF-MS	Approximately 30-50+ protein markers in established high dimensional panels	Ultra-high parameter protein profiling	Very high dimensionality	No live-cell sorting, lower throughput	Deep immune atlas
scRNA-seq	Transcriptome sequencing	Thousands of genes per cell, depending on platform, sequencing depth, sample quality, and analysis pipeline	Gene expression	Molecular state resolution	Limited protein /function information	Cell states and trajectories

Among these approaches, spectral flow cytometry represents an important evolution of fluorescence-based flow cytometry, combining full-spectrum acquisition with computational unmixing to resolve overlapping fluorescence signals and characterize complex immune cell states. From a practical perspective, it offers an accessible approach to high-dimensional immune profiling for laboratories with established fluorescence-based cytometry infrastructure. Compared with mass cytometry, spectral flow cytometry can leverage widely available fluorophore-conjugated antibodies and established cell-handling workflows, while generally offering higher acquisition throughput and compatibility with live-cell analysis and cell sorting when appropriate instrumentation is available. Compared with single-cell RNA sequencing (scRNA-seq), it enables direct protein-level phenotyping without requiring sequencing-based library preparation, although it does not provide the transcriptome-wide molecular information obtainable from sequencing. Nevertheless, spectral flow cytometry requires dedicated instrumentation, appropriate reference controls, optimized panels, and reliable computational unmixing. Its practical accessibility therefore depends on local infrastructure, technical expertise, and experimental objectives. These technologies should be viewed as complementary platforms, with their relative suitability determined by the biological question, required measurement depth, sample characteristics, and available resources. The channel-based detection principle of conventional flow cytometry and its associated limitations, including fluorescence spillover and the need for compensation, are illustrated in **Figure 1A**.

**Figure 1:**
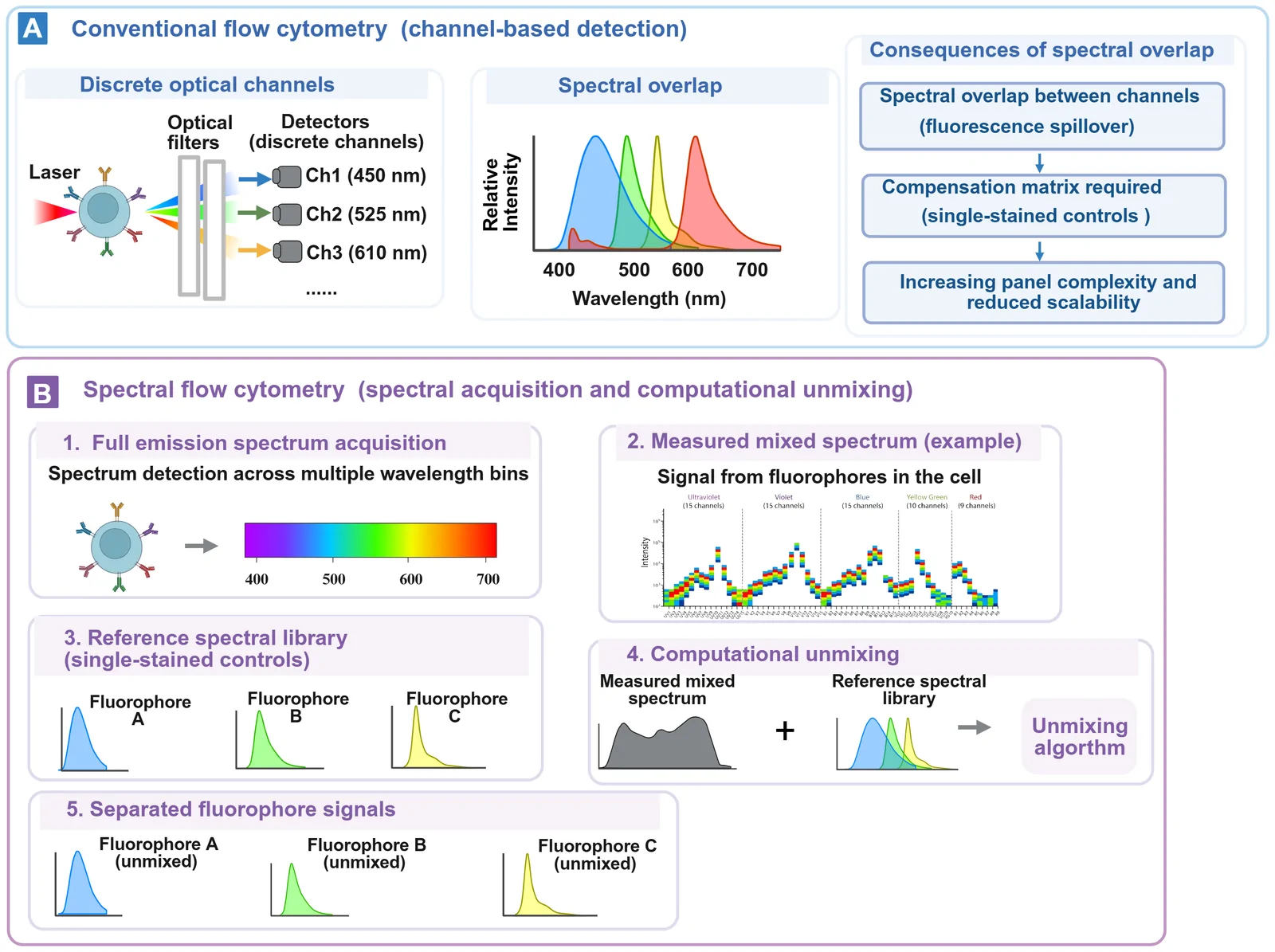
Conceptual transition from conventional flow cytometry to spectral immune profiling. **(A)** Conventional flow cytometry measures fluorescence through discrete optical channels, where spectral overlap between fluorophores produces fluorescence spillover and requires compensation, thereby increasing panel complexity. **(B)** Spectral flow cytometry captures broader emission profiles across the fluorescence spectrum and applies computational unmixing using reference spectral signatures to resolve individual fluorophore contributions from mixed signals.

### 2.2 Spectral acquisition

Unlike conventional flow cytometry, which primarily measures fluorescence intensity within predefined optical channels, spectral flow cytometry captures fluorescence emission characteristics across multiple wavelength ranges to generate comprehensive spectral profiles for each fluorescent label (11). Rather than assigning each fluorophore to an independent detection channel, spectral flow cytometry records the distribution of emitted fluorescence signals across the measured spectrum, providing a multidimensional representation of fluorescence information.

Although different fluorophores may exhibit substantial overlap in their emission spectra, their overall spectral distributions often retain characteristic features that allow computational discrimination. Therefore, each fluorescent label can be represented by a characteristic “spectral fingerprint,” in which subtle differences across multiple wavelength regions contribute to the identification of individual fluorophores (6, 12). This principle transforms spectral overlap from a limitation that must be minimized into a source of information that can be computationally exploited.

The transition from discrete fluorescence intensity measurements to spectral profiling represents a fundamental change in fluorescence-based single-cell analysis. By preserving broader fluorescence signatures rather than relying solely on isolated detector channels, spectral acquisition provides additional information for resolving complex multiparameter panels and expanding the analytical space of high-dimensional immune profiling (13, 14).

This capability is particularly relevant to immunological studies, where cellular identity and functional states are frequently defined by combinatorial expression patterns involving multiple lineage, activation, differentiation, and functional markers. By enabling more comprehensive characterization of these multidimensional phenotypes, spectral acquisition provides an important technological foundation for dissecting immune heterogeneity and cellular states within complex immune ecosystems.

### 2.3 Computational unmixing

Unlike conventional compensation approaches that primarily correct fluorescence spillover between predefined detection channels, spectral unmixing aims to computationally resolve overlapping fluorescence signals based on their complete spectral characteristics. In spectral flow cytometry, the measured signal from each cell is considered a composite spectrum generated by multiple fluorescent components, and computational algorithms are used to estimate the contribution of each fluorophore based on predefined reference spectra (11, 15).

Spectral unmixing typically relies on reference spectral libraries generated from single-stained controls, in which the characteristic emission profile of each fluorophore is experimentally determined. By comparing the acquired cellular spectrum with these reference signatures, the contribution of individual fluorophores can be mathematically estimated and separated from the mixed signal. In addition, autofluorescence components can be incorporated into the unmixing model, which is particularly important for complex biological samples with intrinsic fluorescence backgrounds.

Therefore, the fundamental innovation of spectral flow cytometry is not simply the expansion of detectable parameters, but the transformation of fluorescence overlap from a technical limitation into a computationally interpretable source of information. Through spectral acquisition combined with computational unmixing, spectral flow cytometry enables high-dimensional characterization of immune phenotypes that cannot be fully captured by conventional channel-based fluorescence analysis. Together, spectral acquisition and computational unmixing represent the central technological transition from channel-based fluorescence detection to computationally resolved spectral profiling, providing the basis for high-dimensional immune phenotyping (16, 17) (**Figure 1B**).

### 2.4 High-parameter panel design and experimental quality control

The development of high-parameter spectral flow cytometry panels, particularly those containing 40 or more colors, requires systematic optimization of fluorophore selection, antibody performance, spectral compatibility, and experimental controls (18). Although spectral unmixing allows fluorophores with overlapping emission profiles to be used within the same panel, this flexibility does not eliminate the effects of spectral similarity or spillover spreading. Panel design should therefore begin with the biological objectives of the experiment, including the target cell populations, expected antigen expression levels, marker co-expression patterns, and the resolution required to distinguish closely related cellular states. Bright fluorophores should generally be prioritized for low-abundance antigens or markers with weak expression, whereas highly expressed antigens may be assigned less bright fluorophores, provided that adequate resolution is maintained. Fluorophore combinations should also be evaluated in the context of the specific instrument configuration and the anticipated co-expression of their corresponding markers. In silico panel-design tools can help identify potentially problematic fluorophore combinations before experimental testing, but computational predictions should be followed by empirical validation using the intended samples and staining conditions (18, 19).

Two useful metrics for evaluating spectral panel design are the similarity index (SI) and the complexity index (CI) (19, 20). The SI describes the spectral similarity between a pair of fluorophores, whereas the CI provides an aggregate measure of the spectral complexity of a proposed panel. Higher spectral similarity may make fluorophores more difficult to distinguish during unmixing, while greater panel complexity may indicate an increased risk of spreading-related resolution loss. These metrics can support fluorophore selection and marker assignment, but they should not be interpreted as direct evidence that a panel will perform adequately in biological samples. Their interpretation depends on the instrument configuration and the reference spectra used, and acceptable theoretical metrics do not replace experimental assessment of marker separation, background signals, and population resolution. Accordingly, high-parameter panels should be evaluated iteratively, with particular attention to dim markers, co-expressed antigens, rare populations, and phenotypically similar cell subsets.

Appropriate controls are essential for validating the complete spectral workflow. Single-stained reference controls are used to establish the spectral signatures required for unmixing and should adequately represent the fluorophore conjugates used in the experiment. Unstained controls are important for characterizing background fluorescence and assessing autofluorescence, particularly in heterogeneous or tissue-derived samples. Fluorescence-minus-one (FMO) controls, in which one antibody or fluorophore is omitted from an otherwise complete staining panel, help establish gating boundaries for markers with dim, continuous, or overlapping expression patterns. Additional controls should be selected according to the experimental objectives and may include viability controls, biological positive and negative controls, and appropriate controls for intracellular staining or functional stimulation. These controls serve different purposes and should not be considered interchangeable (21).

Validation should extend beyond successful computational unmixing of single-stained reference controls. The fully stained sample should also be examined to assess residual background signals, unexpected population distributions, spreading-related loss of resolution, and the separation of relevant positive and negative populations. Antibody titration, instrument performance monitoring, consistent sample preparation, and systematic evaluation of the final gating strategy are particularly important as panel complexity increases. Where feasible, pilot experiments and replicate samples should be used to determine whether the intended populations can be reproducibly resolved. For longitudinal or multicenter studies, the experimental design should additionally document the instrument configuration, fluorophore–antibody combinations, reference controls, unmixing procedures, relevant quality-control results, and analytical settings. Together, these measures help distinguish theoretical panel compatibility from demonstrated experimental performance and improve the reproducibility of high-dimensional immune profiling (18, 21).

### 2.5 Advantages and current limitations of spectral flow cytometry

#### 2.5.1 Advantages

##### 2.5.1.1 Enabling high-dimensional immune state profiling

Spectral flow cytometry expands fluorescence-based single-cell analysis by enabling the simultaneous characterization of multiple phenotypic and functional markers within individual cells (9). Unlike conventional flow cytometry, which relies on predefined detection channels and compensation strategies, spectral flow cytometry captures multidimensional fluorescence information that is particularly valuable for resolving heterogeneous biological systems, including immune ecosystems and tumor microenvironments (12, 22, 23). By integrating lineage, differentiation, activation, and functional markers within a single analytical framework, spectral flow cytometry facilitates comprehensive characterization of cellular composition and immune states at the single-cell level. The resulting multidimensional information supports the characterization of diverse immune cell populations, the resolution of distinct cellular states, and the mapping of complex immune ecosystems (**Figure 2**).

**Figure 2:**
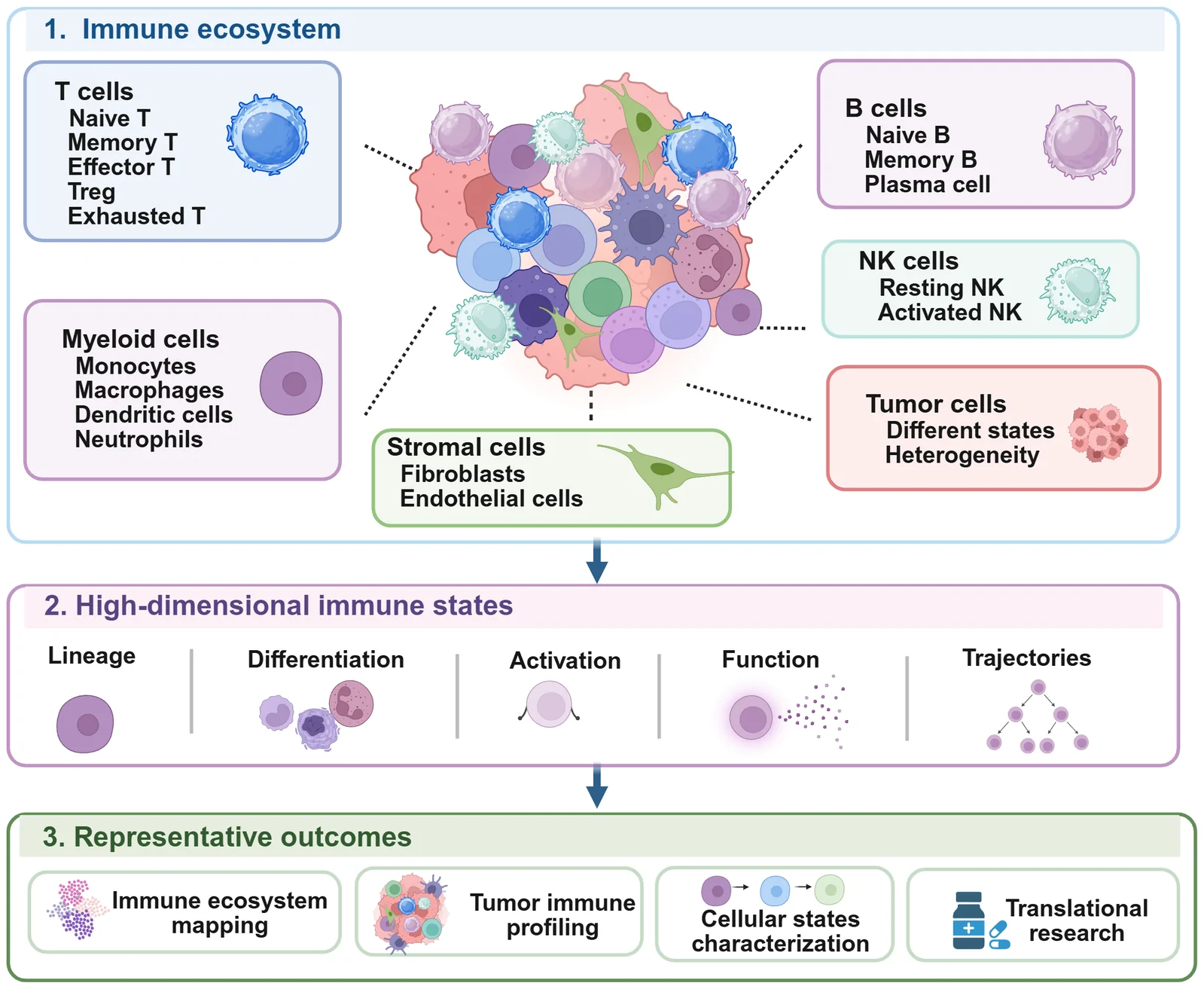
From spectral information to high-dimensional immune state profiling. Spectral flow cytometry enables multidimensional characterization of immune ecosystems by resolving diverse immune cell populations and their differentiation, activation, and functional states. This information supports immune ecosystem mapping, tumor immune profiling, cellular state characterization, and translational research.

##### 2.5.1.2 Increasing flexibility in multiparameter panel design

Spectral flow cytometry also expands the conceptual framework of multiparameter panel design. Rather than relying primarily on minimizing spectral overlap between fluorophores, spectral analysis incorporates complete emission profiles into computational unmixing, thereby relaxing some of the constraints imposed by conventional channel-based detection (6).

This increased flexibility allows panel design to place greater emphasis on biological objectives, including antigen expression levels, marker combinations, and cellular-state resolution, rather than solely on minimizing fluorescence interference (24). Consequently, spectral flow cytometry provides greater opportunities to construct high-dimensional panels tailored to complex biological questions.

##### 2.5.1.3 Resolving rare and functionally distinct cellular states

The simultaneous measurement of multiple phenotypic dimensions within individual cells facilitates the identification of rare populations and functionally distinct cellular states that may be difficult to resolve using conventional lower-dimensional approaches (25). This capability is particularly relevant to studies of tumor immune microenvironments, autoimmune diseases, infectious diseases, and minimal residual disease, where biologically important populations may be defined by complex combinations of markers rather than by a single lineage-associated antigen (26–28).

Importantly, spectral flow cytometry should not be interpreted as a universal increase in analytical sensitivity. The reliable detection of rare populations and low-abundance markers remains dependent on antibody quality, staining optimization, instrument performance, background fluorescence, and downstream analytical strategies. Thus, its principal advantage lies in expanding multidimensional information content and cellular-state resolution, rather than simply increasing detection sensitivity.

Collectively, these features establish spectral flow cytometry as more than a higher-parameter extension of conventional flow cytometry. By combining full-spectrum fluorescence acquisition with computational analysis, it provides a flexible platform for resolving complex immune heterogeneity and multidimensional cellular states.

#### 2.5.2 Current limitations

Despite these advantages, several technical and analytical limitations currently constrain the reproducibility and broader implementation of spectral flow cytometry. First, reliable spectral unmixing depends on the quality of reference spectra, instrument performance, staining consistency, and sample preparation, creating potential variability across experimental batches, instruments, and laboratories (3, 29, 30). Second, autofluorescence remains an important consideration in tissue-derived and highly autofluorescent samples, where endogenous fluorescence may complicate the separation of target signals from biological background (7, 31, 32).

The high dimensionality of spectral datasets also introduces challenges in data preprocessing, computational analysis, and biological interpretation. Although dimensionality reduction, clustering, machine learning, and artificial intelligence approaches can facilitate the analysis of complex datasets, differences in analytical workflows may affect reproducibility and interpretation (15, 25). Finally, broader clinical adoption requires standardized analytical workflows, validated performance criteria, and reproducible implementation across laboratories (9, 23, 33).

These limitations do not diminish the utility of spectral flow cytometry but highlight the methodological requirements that must be addressed as the technology moves from research-oriented high-dimensional profiling toward standardized and clinically applicable immune analysis. The major challenges and emerging strategies for addressing these issues are discussed in greater detail in Section 5.

## 3 Decoding immune heterogeneity with spectral flow cytometry

The transition from conventional marker-based immunophenotyping to spectral profiling has expanded the scope of single-cell immune analysis from predefined cellular classification toward multidimensional characterization of immune ecosystems. By integrating high-dimensional fluorescence acquisition with computational unmixing, spectral flow cytometry enables simultaneous assessment of immune composition, cellular states, functional programs, and disease-associated alterations across diverse biological contexts (**Figure 3**). These capabilities provide a framework for mapping immune ecosystems in health and disease, resolving tumor-associated immune heterogeneity and therapeutic responses, tracing immune development and cellular fate, and translating high-dimensional immune profiles into clinically relevant biomarkers and therapeutic insights.

**Figure 3:**
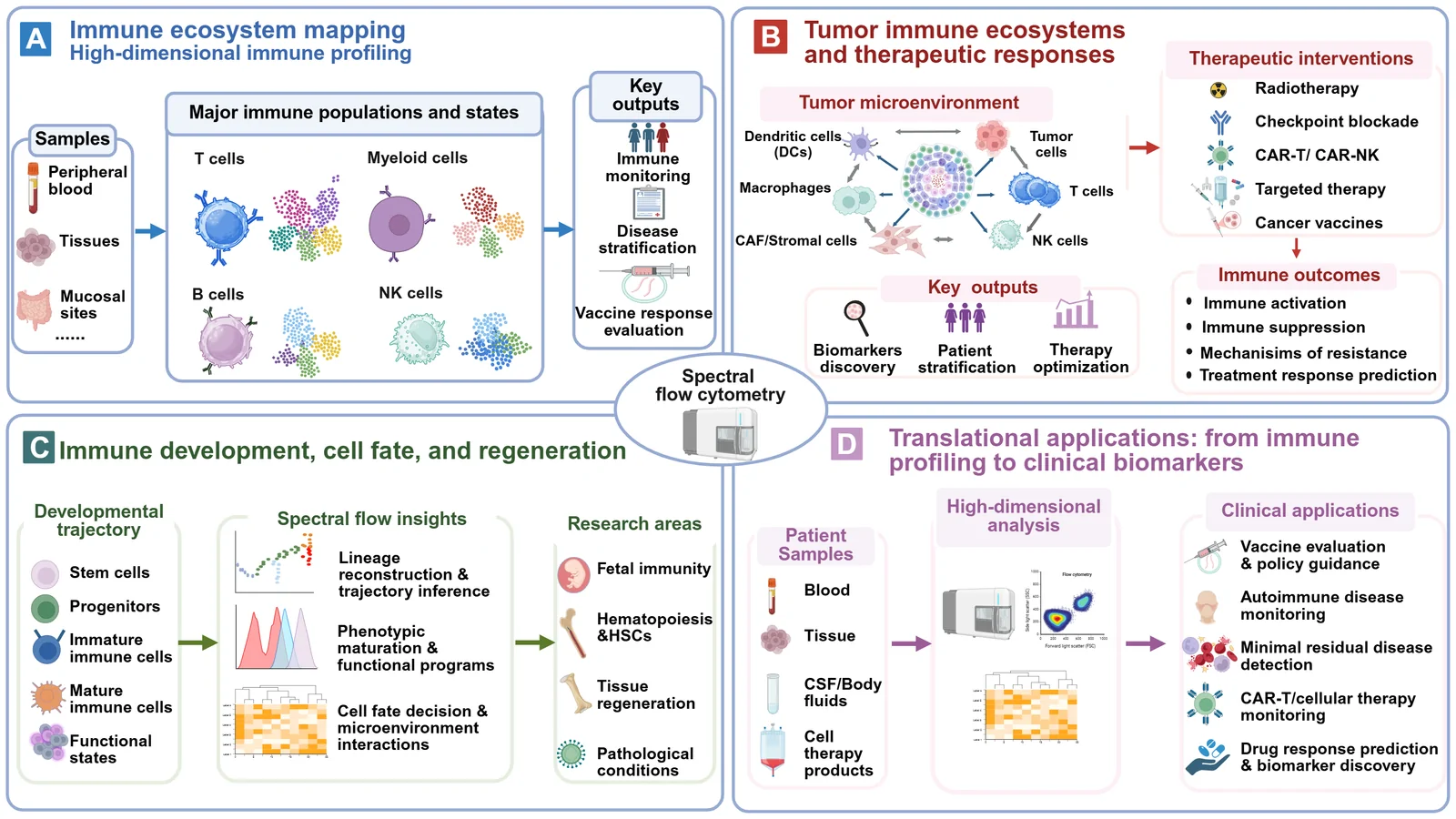
Biological applications of spectral flow cytometry in immune research and translational medicine. **(A)** Spectral flow cytometry enables high-dimensional immune ecosystem mapping by simultaneously resolving immune cell composition, functional states, and disease-associated immune alterations. **(B)** In cancer immunology, spectral profiling reconstructs tumor immune ecosystems and identifies cellular interactions, therapeutic response signatures, and resistance mechanisms. **(C)** Spectral analysis facilitates investigation of immune development, cellular differentiation trajectories, hematopoietic regulation, and tissue regeneration. **(D)** Translational applications include vaccine evaluation, autoimmune monitoring, minimal residual disease detection, cellular therapy assessment, and biomarker discovery. Collectively, these applications highlight the potential of spectral flow cytometry to advance multidimensional immune profiling and precision immunology.

### 3.1 Immune ecosystem mapping

#### 3.1.1 From immune cell identification to multidimensional immune ecosystem profiling

The immune system is not composed of isolated cellular populations but represents a highly interconnected network of immune subsets with distinct developmental origins, functional programs, and regulatory interactions. Conventional flow cytometry has long served as a cornerstone technology for immune phenotyping by enabling the identification of major immune lineages based on predefined marker combinations. However, as immunological research has shifted from simple cell classification toward comprehensive characterization of cellular states, conventional approaches have become increasingly insufficient for capturing the dynamic transitions associated with immune activation, differentiation, exhaustion, and functional adaptation.

Spectral flow cytometry provides a new framework for systematic immune ecosystem profiling by integrating full-spectrum acquisition, high-dimensional fluorescence detection, and computational unmixing. By overcoming the limitations imposed by predefined optical channels, spectral flow cytometry enables simultaneous assessment of lineage markers, differentiation states, functional molecules, and activation programs at the single-cell level. This technological transition shifts immune analysis from identifying “which cells are present” toward understanding “what functional states these cells represent,” thereby enabling more comprehensive reconstruction of immune landscapes.

#### 3.1.2 Standardized high-dimensional immune profiling across blood and tissues

A major advancement in spectral flow cytometry has been the establishment of standardized high-dimensional immune profiling platforms. Several optimized multicolor panels have been developed to enable comprehensive characterization of human immune populations. These include the 40-color OMIP-069 reference panel, subsequent 45-color expansions, and the highly multiplexed 50-color OMIP-102 panel, which collectively enable deep phenotyping of major immune compartments in peripheral blood, including T cells, B cells, natural killer (NK) cells, and myeloid populations, while further resolving memory, activated, exhausted, and functionally specialized subsets (34–36).

Beyond standardized peripheral blood profiling, spectral flow cytometry-based approaches have been further adapted for disease-specific and tissue-specific immune investigations. Specialized panels have been developed for acute myeloid leukemia, intestinal lymphocyte characterization, and immune profiling in experimental models including liver tissues, colorectal cancer models, and brain metastasis settings (37–41). Furthermore, the extension of these analytical frameworks to additional species, including rodents and non-human primates, has expanded their utility for translational immunology research and comparative immune studies (42, 43).

These standardized platforms have transformed spectral flow cytometry from an advanced analytical technology into a reproducible framework for immune monitoring across diverse biological contexts.

#### 3.1.3 Mapping immune states in health and disease

The biological value of spectral flow cytometry extends beyond increasing the number of measurable parameters. Its major contribution lies in the ability to resolve subtle immune states and dynamic changes that cannot be fully captured by conventional low-dimensional approaches. In complex diseases, immune dysfunction often results from coordinated alterations across multiple cellular compartments, requiring simultaneous evaluation of immune composition, activation status, and functional characteristics.

During the COVID-19 pandemic, spectral flow cytometry was widely applied to characterize immune remodeling associated with infection. By integrating multiple immune parameters within individual cells, these studies revealed dynamic alterations in immune composition and functional states, providing insights into mechanisms underlying immune dysregulation during disease progression (44). Similarly, in autoimmune disorders such as granulomatosis with polyangiitis, high-dimensional immune profiling has contributed to the identification of disease-associated immune signatures and cellular characteristics linked to disease activity, supporting the development of more precise immune monitoring strategies (45).

Compared with conventional immune profiling, spectral flow cytometry enables a transition from descriptive enumeration of immune populations toward functional interpretation of immune ecosystems. This capability is particularly important for investigating diseases characterized by immune heterogeneity, including infection, autoimmune inflammation, cancer, and immune-mediated disorders.

#### 3.1.4 Toward systems-level reconstruction of immune landscapes

With the continuous expansion of high-dimensional detection capabilities, spectral flow cytometry is facilitating a transition from static immune phenotyping toward systems-level reconstruction of immune landscapes. By simultaneously analyzing immune composition, functional states, and potential interactions among cellular populations, spectral approaches provide a comprehensive framework for understanding immune organization in physiological and pathological conditions.

Despite these advances, analytical standardization, cross-platform reproducibility, and the biological interpretation of high-dimensional datasets remain important challenges. Integrating spectral flow cytometry with single-cell transcriptomics, spatial omics, and computational approaches may help address some of these limitations, although the complementary contributions and technical constraints of each platform must be considered.

Critical synthesis and remaining challenges. Collectively, these studies demonstrate that the principal contribution of spectral flow cytometry to immune ecosystem mapping lies not simply in increasing the number of measurable markers, but in enabling the simultaneous characterization of immune composition, differentiation, activation, and functional states within a unified single-cell framework. Standardized high-dimensional panels provide a foundation for systematic immune profiling, whereas disease-specific and tissue-adapted panels allow investigators to address biological questions that require more specialized marker combinations. However, these approaches are complementary rather than universally interchangeable. Differences in tissue composition, sample processing, panel design, marker selection, and analytical workflows can influence the immune populations resolved and the biological conclusions drawn. Moreover, the identification of multidimensional phenotypic patterns does not, by itself, establish cellular function, causal interactions, or spatial organization within an immune ecosystem. Accordingly, reliable immune ecosystem mapping requires biologically informed panel design, appropriate experimental controls, reproducible computational analysis, and validation of key findings using complementary functional or molecular approaches. These considerations are particularly important when comparing results across diseases, tissues, cohorts, and experimental platforms. Integrating spectral flow cytometry with complementary single-cell and spatial technologies may further strengthen biological interpretation, provided that technical variability and the distinct information contributed by each platform are explicitly considered.

### 3.2 Decoding tumor immune ecosystems and therapeutic responses

#### 3.2.1 High-dimensional reconstruction of tumor immune ecosystems

Tumor progression and therapeutic response are shaped by complex interactions among malignant cells, immune populations, and stromal components within the tumor microenvironment (TME). However, conventional immune profiling approaches often rely on limited marker combinations and predefined cellular classifications, which may fail to capture the functional diversity and dynamic states of tumor-associated immune populations. Spectral flow cytometry provides a high-dimensional strategy for reconstructing tumor immune ecosystems by simultaneously resolving immune lineage, activation status, functional programs, and cellular interactions at the single-cell level.

By integrating full-spectrum acquisition with computational unmixing, spectral flow cytometry enables comprehensive characterization of tumor-associated immune landscapes beyond conventional immunophenotyping. In breast cancer leptomeningeal metastasis, spectral profiling of cerebrospinal fluid and peripheral immune compartments revealed distinct alterations in T-cell subsets and monocyte populations, providing insights into immune characteristics associated with this challenging disease setting (46). In renal cell carcinoma, spectral analysis demonstrated that radiotherapy enhanced PD-1^+^ T-cell infiltration and increased tumor–immune interactions, providing mechanistic evidence supporting combined radiotherapy and immunotherapy strategies (47). Similarly, in malignant peripheral nerve sheath tumors, high-dimensional immune profiling revealed that targeted therapy combined with oncolytic viruses promoted activation of germinal center B cells and cytokine-producing CD4^+^ T cells, suggesting potential strategies for improving therapeutic responses (48).

Collectively, these studies demonstrate the value of spectral flow cytometry in characterizing tumor immune heterogeneity across different disease and treatment settings. However, the immune features identified may vary with tumor type, tissue source, and panel design, highlighting the importance of context-specific interpretation and complementary validation.

#### 3.2.2 Defining immune determinants of therapeutic response and resistance

Beyond describing tumor immune composition, spectral flow cytometry has enabled identification of immune features associated with therapeutic response and resistance. In acute myeloid leukemia, analysis of donor lymphocyte infusion products demonstrated that effector T-cell abundance and exhaustion-associated states correlated with relapse risk, suggesting potential biomarkers for predicting treatment outcomes (49). In chronic lymphocytic leukemia, spectral profiling revealed abnormal CD8^+^ T-cell expansion associated with targeted therapy resistance, indicating that immune dysregulation beyond malignant B-cell targeting contributes to treatment failure (50).

Furthermore, spectral approaches have facilitated the discovery of specialized immune populations involved in antitumor immunity, including CD169^+^ macrophages with enhanced antitumor activity through interaction with CD8^+^ T cells (51), as well as tissue-resident memory CD8^+^ T cells and classical type 1 dendritic cells contributing to melanoma immune protection (52).

These findings highlight the importance of assessing immune phenotypes when investigating therapeutic response and resistance. However, associations between immune features and treatment outcomes do not necessarily establish causality, underscoring the need for independent-cohort validation and functional studies.

#### 3.2.3 Spectral flow cytometry in biomarker discovery and clinical monitoring

A major translational value of spectral flow cytometry lies in its ability to support biomarker discovery and therapeutic monitoring. High-dimensional immune profiling has identified regulatory targets involved in tumor immunity through modulation of T-cell infiltration, macrophage polarization, and interferon signaling pathways (53–56).

Integration of spectral flow cytometry with single-cell transcriptomic approaches has further revealed lineage-associated immune and malignant cell states, such as lineage-biased patterns in NPM1-mutated acute myeloid leukemia, providing a biological basis for subtype-specific therapeutic strategies (57).

In clinical monitoring settings, ultra-high-parameter spectral panels have enabled minimal residual disease detection with sensitivities approaching 0.1% and allowed simultaneous assessment of leukemia stem cell characteristics and sample quality, supporting integration into standardized workflows (58–61). In cellular immunotherapy, 37-parameter spectral profiling identified PD-1 expression on CD8^+^ CAR-T cells as a predictor of complete response, while integration with single-cell sequencing revealed FOXO1 as a regulator of CAR-T memory differentiation and antitumor activity (62, 63). Additional studies identified immune signatures associated with CAR-T resistance, including expansion of double-negative T cells expressing multiple co-inhibitory molecules in refractory multiple myeloma (64).

These applications highlight the potential of spectral flow cytometry in biomarker discovery and clinical monitoring. Nevertheless, analytical performance and clinical predictive value require separate validation, supported by standardized procedures and independent patient cohorts.

#### 3.2.4 Guiding next-generation immunotherapy development

Spectral flow cytometry has also contributed to understanding mechanisms underlying emerging combination immunotherapies. Combined CAR-NK cell therapy and systemic PD-1 blockade enhanced infiltration of CD4^+^ T cells and NKT-like cells, thereby reversing immunosuppressive tumor microenvironments (65). Similarly, PD-L1/TGF-β bispecific antibody therapy combined with chemotherapy reshaped immunologically cold pancreatic tumors by enhancing effector immune populations while reducing suppressive immune subsets (66).

Moreover, spectral flow-based immune profiling has supported preclinical evaluation of emerging therapeutic platforms, including macrophage–drug conjugates and nanotechnology-based immunotherapies that promote cytotoxic lymphocyte infiltration, IFN-γ signaling activation, and macrophage polarization toward antitumor states (67, 68).

Collectively, spectral flow cytometry has transformed tumor immunology from static immune-cell enumeration toward multidimensional ecosystem-level analysis, linking immune composition, cellular states, and therapeutic responses. This capability provides a powerful framework for biomarker discovery, patient stratification, and precision immunotherapy development. However, differences in sample processing, panel design, and analytical workflows may limit cross-study comparability, highlighting the need for standardized procedures and independent validation before clinical implementation.

### 3.3 Immune development, cell fate, and regeneration

#### 3.3.1 Mapping immune development and cellular differentiation trajectories

Immune development is a highly dynamic process involving continuous transitions in cellular identity, differentiation programs, and functional maturation. Conventional flow cytometry has traditionally characterized immune development using predefined lineage markers; however, such approaches provide limited resolution for capturing transitional states and developmental trajectories. Spectral flow cytometry enables multidimensional characterization of developing immune populations by simultaneously integrating lineage markers, maturation-associated molecules, and functional states at the single-cell level.

During early human development, spectral flow cytometry has provided unprecedented insights into the establishment of immune ecosystems. A 25-color spectral flow cytometry strategy was applied to characterize immune development in the mid-gestation human fetal intestine, revealing unique immune features characterized by enrichment of CD4^+^ T cells and innate lymphoid cells with relatively limited cytokine production, providing insights into early immune tolerance establishment (69). Combined with multi-omics approaches, spectral profiling further identified a conserved fetal intestinal microenvironment supporting local B-cell development, challenging previous assumptions regarding early mucosal immune organization (70).

Together, these studies demonstrate the value of spectral flow cytometry in resolving developmental immune populations that may be overlooked by conventional lineage-based profiling. However, phenotypic differences alone cannot fully establish developmental trajectories or functional roles, highlighting the importance of complementary molecular and functional validation.

#### 3.3.2 Resolving hematopoietic stem cell differentiation and immune reconstitution

Beyond immune development, spectral flow cytometry has facilitated detailed analysis of hematopoietic stem and progenitor cell heterogeneity. High-dimensional panels containing 20–22 parameters have been developed to characterize hematopoietic stem/progenitor populations and define phenotypic continua during fetal hematopoiesis (71, 72).

Following transplantation, integration of high-parameter spectral flow cytometry with sequencing approaches enabled longitudinal tracking of NK-cell receptor repertoire reconstruction and revealed the impact of cytomegalovirus infection on immune reconstitution (73). Furthermore, spectral profiling demonstrated that alcohol exposure can reshape hematopoietic differentiation trajectories by promoting skewing toward osteoclast lineage commitment, providing mechanistic insights into alcohol-associated bone loss (74).

These findings highlight the potential of spectral flow cytometry to characterize hematopoietic heterogeneity and monitor immune reconstitution under different physiological and pathological conditions. Nevertheless, reliable interpretation of developmental changes requires consideration of sampling time, biological variability, and complementary evidence to distinguish phenotypic associations from underlying mechanisms.

#### 3.3.3 Defining regeneration-associated progenitor states

Spectral flow cytometry has also expanded the understanding of tissue regeneration by identifying functionally distinct progenitor populations within regenerative niches. In skeletal regeneration, spectral profiling of different bone compartments identified a CD90^+^CD34^+^ stem/progenitor population enriched in the periosteum, suggesting potential cellular targets for bone repair strategies (75).

In muscle biology, spectral flow cytometry resolved heterogeneous fibro-adipogenic progenitor populations and identified DLK1 as a key regulator of cell fate decisions, providing potential therapeutic insights into muscle degeneration and regeneration (76). Under pathological conditions, spectral analysis further revealed disease-associated progenitor expansion, including increased CD143^+^ vascular progenitor-like populations in severe COVID-19 patients, suggesting their potential involvement in pathological angiogenesis (77).

Collectively, these studies demonstrate that spectral flow cytometry extends immune analysis beyond endpoint characterization toward dynamic reconstruction of developmental trajectories and cellular fate decisions. By resolving transitional immune states and progenitor heterogeneity, this technology provides a powerful framework for understanding regeneration, immune maturation, and tissue adaptation. However, differences in sample preparation, panel design, and analytical strategies may affect the populations identified across studies, while functional validation remains essential for establishing their biological significance and regenerative potential.

### 3.4 Translational applications: from immune profiling to clinical biomarkers

#### 3.4.1 Spectral profiling of vaccine-induced immune responses

Vaccination represents one of the most important clinical scenarios in which immune heterogeneity determines the magnitude, durability, and quality of protective responses. However, conventional immune monitoring approaches based on limited marker combinations often fail to capture the complex cellular interactions underlying vaccine-induced immunity. Spectral flow cytometry provides a high-dimensional platform for simultaneously resolving immune cell composition, activation states, and functional characteristics, enabling comprehensive evaluation of vaccine responses at the single-cell level.

During the COVID-19 pandemic, spectral flow cytometry played an important role in dissecting immune mechanisms induced by different vaccine platforms. By simultaneously tracking germinal center B-cell responses, plasmablast differentiation, follicular helper T-cell populations, and CD11c^+^ B-cell subsets, spectral profiling provided detailed insights into the cellular programs associated with mRNA vaccine-induced immunity (78–82). Comparative analyses between mRNA and adenoviral vector vaccines further demonstrated distinct immune response patterns, revealing stronger immunogenicity associated with mRNA vaccination and providing evidence for optimizing booster strategies (83, 84).

Beyond vaccine platform comparison, spectral flow cytometry has contributed to the evaluation of vaccination strategies, including sequential immunization approaches, heterologous booster regimens, and alternative administration routes (85, 86). Importantly, high-dimensional immune profiling has revealed how baseline immune states influence vaccine responsiveness in specific populations, including individuals with previous hepatitis C virus clearance and diverse ethnic backgrounds, highlighting the importance of personalized vaccination strategies (87, 88).

In pneumococcal vaccine development, spectral flow cytometry enabled comprehensive characterization of polysaccharide-specific B-cell responses, providing valuable information for the optimization of next-generation conjugate vaccines (89). Furthermore, in cancer vaccine studies, spectral profiling has facilitated multidimensional evaluation of vaccine efficacy by simultaneously assessing multifunctional T-cell responses, epitope spreading, and memory/exhaustion states of T and NK cells (90). In preclinical studies of intraperitoneal cancer vaccines, spectral analysis further demonstrated that localized immune activation could induce systemic antitumor immunity, supporting the development of novel vaccination strategies for metastatic cancer (91).

Collectively, these studies establish spectral flow cytometry as a critical bridge between immune mechanism discovery and translational vaccine development by enabling comprehensive characterization of vaccine-induced immune landscapes. However, vaccine-induced immune phenotypes do not necessarily translate directly into protective immunity or long-term clinical efficacy. Integrating cellular profiling with functional immune assays and clinical outcomes is therefore important for validating candidate correlates of protection and informing vaccine optimization.

#### 3.4.2 High-dimensional immune profiling for autoimmune biomarkers and disease stratification

Autoimmune diseases are characterized by substantial immunological heterogeneity, in which distinct immune cell states contribute differently to disease initiation, progression, and therapeutic response. Traditional immune profiling approaches often provide limited information regarding functional diversity within immune populations. Spectral flow cytometry has facilitated a transition from descriptive immune phenotyping toward mechanistic characterization and biomarker discovery in autoimmune disorders.

In rheumatoid arthritis, spectral profiling identified B-cell populations capable of recognizing multiple post-translationally modified antigens, revealing previously unappreciated diversity in autoreactive immune responses (92). Similarly, high-dimensional immune analysis has identified disease-associated cellular populations, including SAP^+^ T-cell populations associated with lupus nephritis in systemic lupus erythematosus (SLE) and atypical B-cell subsets in myasthenia gravis, providing new insights into disease heterogeneity and immune classification (93, 94).

Longitudinal spectral flow analyses have further revealed dynamic immune trajectories associated with autoimmune disease progression. In rheumatoid arthritis, changes in citrullinated antigen-specific T-cell frequencies were associated with disease evolution. In SLE, multidimensional immune profiling delineated an interferon-driven pathway involving memory B-cell activation, plasmablast differentiation, and antibody production. In systemic sclerosis and Sjögren’s syndrome, spectral analysis demonstrated combined exhaustion and metabolic dysfunction of T cells associated with interstitial lung disease development (95–97).

Beyond immune characterization, spectral flow cytometry has contributed to understanding therapeutic mechanisms and identifying potential intervention targets. Studies in primary Sjögren’s syndrome demonstrated that antibody-secreting cell maturation states could serve as quantitative biomarkers and potential therapeutic targets (98). In lupus, spectral profiling identified IL-16 as a key regulator promoting T-cell tissue infiltration, suggesting new therapeutic opportunities (99). Furthermore, analysis of anti-CD20 therapy revealed that B-cell depletion is accompanied by modulation of inflammatory IL1B-expressing monocyte populations, expanding the understanding of immune regulation mediated by B-cell-targeted therapies (100).

Together, these findings demonstrate that spectral flow cytometry provides a powerful framework for autoimmune disease stratification, biomarker discovery, and mechanism-based therapeutic development. These studies highlight the potential of spectral flow cytometry to distinguish immunologically diverse patient subgroups and identify candidate biomarkers of disease activity and therapeutic response. However, disease-associated immune signatures may vary with disease stage, treatment history, and patient characteristics, requiring independent-cohort validation before their use in clinical stratification.

#### 3.4.3 From immune profiling to therapeutic discovery and disease modeling

Beyond clinical immune monitoring, spectral flow cytometry has increasingly contributed to therapeutic discovery, drug evaluation, and disease modeling by enabling comprehensive assessment of immune responses in complex experimental systems.

In drug discovery, high-dimensional spectral flow platforms have been developed for evaluating the effects of candidate compounds across major immune populations. A dual 22-color spectral profiling strategy enabled simultaneous assessment of immune cell composition and activation states, demonstrating the potential of spectral flow cytometry as a high-throughput approach for immunomodulatory drug screening (101). Furthermore, spectral analysis revealed mechanisms of action for therapeutic compounds, including selective inhibition of proliferative responses without substantial effects on non-malignant cells, supporting the development of more targeted treatment strategies (102).

Spectral flow cytometry has also enhanced disease modeling by enabling molecular and cellular validation of advanced experimental systems. In pituitary tumor organoid models derived from Cushing disease, spectral profiling demonstrated similarities between organoids and primary tumors at the protein-expression level, supporting their application in disease modeling and personalized drug testing (103). Similarly, spectral analysis of melanoma tumor spheroids validated key immune and tumor-associated markers, establishing a platform integrating organoid technology with high-throughput therapeutic screening (104).

High-dimensional immune profiling has further facilitated biomarker discovery in inflammatory and infectious diseases. Combined analysis of immune phenotypes, cytokine production, and clustering approaches identified Gal-9 and soluble Tim-3 as potential early diagnostic biomarkers for unexplained acute hepatitis in children (105). In sepsis, spectral analysis revealed the EGFR/AREG pathway as a regulator of T-cell dysfunction, identifying AREG as both a prognostic biomarker and a potential immunomodulatory target (106).

Moreover, spectral flow cytometry has expanded understanding of immunometabolic regulation by linking immune phenotypes with functional metabolic states. High-parameter profiling revealed metabolic heterogeneity among tissue-resident macrophages during homeostasis and infection (107), and demonstrated distinct metabolic programs associated with functionally specialized pulmonary T-cell populations, providing insights for metabolically targeted vaccine strategies (108).

In metabolic and infectious diseases, spectral flow cytometry has enabled detailed characterization of immune dysfunction. Studies in diabetes demonstrated altered T-cell composition and expansion of inflammatory Th1/Th17 populations associated with impaired immune defense against Mycobacterium tuberculosis (109, 110). In infectious disease settings, spectral approaches have characterized pathogen-specific immune responses, including tuberculosis-specific Th17 responses influenced by HIV infection and kynurenine pathway activation (111), long-term alterations of innate-like T-cell function following HIV exposure (112, 113), and γδ T-cell remodeling during chronic treated infection (114, 115). Additionally, spectral profiling revealed pregnancy-associated immune tolerance mechanisms during Zika virus infection and enabled evaluation of circulating T follicular helper responses to malaria vaccine candidates (116, 117).

Collectively, these applications demonstrate that spectral flow cytometry has expanded beyond immune phenotyping toward a broader translational platform integrating biomarker discovery, therapeutic evaluation, and disease modeling. Representative applications of spectral flow cytometry across immune profiling, disease modeling, therapeutic evaluation, and clinical translation are summarized in **Table 2**. However, the translation of these applications into clinical practice requires standardized analytical workflows, independent validation, and confirmation of the biological and clinical relevance of candidate biomarkers.

**Table 2:** Representative applications of spectral flow cytometry in immune research and translational medicine.

Biological context	Scientific question	Key immune insights enabled by spectral flow cytometry	Representative examples
Immune ecosystem mapping	How immune states are organized?	Comprehensive profiling of immune subsets, activation, exhaustion and functional states	COVID-19, autoimmune diseases
Tumor immune ecosystem	How immune cells regulate tumor progression and therapy response?	Identification of immune biomarkers, resistance mechanisms and therapeutic targets	RCC, AML, melanoma
Vaccine immunology	How vaccines induce protective immunity?	Tracking GC B cells, Tfh cells, memory responses	mRNA vaccines
Development and regeneration	How immune cells differentiate and mature?	Mapping developmental trajectories and progenitor states	Fetal immunity, HSC
Cellular therapy	What determines therapeutic efficacy?	CAR-T phenotype, exhaustion, persistence	CAR-T studies
Clinical translation	Can immune profiles become biomarkers?	MRD detection, patient stratification	Leukemia monitoring

## 4 Integrating spectral flow cytometry into multimodal immune profiling

### 4.1 Complementarity with mass cytometry: expanding and validating high-dimensional immune analysis

The emergence of spectral flow cytometry and mass cytometry has significantly expanded the capacity of single-cell immune profiling beyond conventional fluorescence-based approaches. Although these two platforms differ fundamentally in signal detection principles, they should not be viewed as competing technologies but rather as complementary strategies for high-dimensional immune analysis.

Cross-platform validation studies have demonstrated that antibody panels optimized for high-dimensional immune profiling can generate highly concordant cellular classifications across spectral flow cytometry and mass cytometry platforms, supporting the reproducibility of complex immune phenotyping and enabling robust comparison of rare immune populations across experimental settings (118).

Beyond methodological validation, combined application of spectral flow cytometry and mass cytometry provides complementary advantages in biological and clinical studies. Mass cytometry offers exceptional parameter capacity through metal isotope labeling, whereas spectral flow cytometry preserves the advantages of fluorescence-based analysis, including live-cell sorting capability and compatibility with functional assays. Their integration has facilitated comprehensive immune profiling in autoimmune diseases, hematological malignancies, and drug toxicity studies, generating multidimensional evidence for biomarker discovery and disease mechanism investigation (119–121).

Together, these approaches represent complementary components of next-generation cytometry platforms, where ultra-high-dimensional measurement and functional immune analysis can be integrated.

### 4.2 Artificial intelligence and computational frameworks for high-dimensional immune interpretation

The increasing dimensionality of spectral flow cytometry datasets has created opportunities for artificial intelligence (AI) and machine learning (ML) to support both experimental design and biological interpretation. However, the value of these approaches extends beyond the automated analysis of large datasets. Their potential contributions span panel optimization, immune population identification, cellular-state annotation, and the development of predictive models. At the same time, their reliability depends on appropriate experimental design, transparent analytical procedures, and validation across independent datasets.

AI-assisted panel design and experimental optimization. High-parameter spectral flow cytometry panels require careful consideration of fluorophore brightness, spectral similarity, antigen abundance, marker co-expression, and the biological objectives of the study. Computational and algorithm-assisted design approaches can help evaluate candidate fluorophore-antibody combinations, identify potentially problematic spectral overlaps, and prioritize marker assignments before experimental testing. Such approaches may reduce the number of trial-and-error iterations required during panel development. Nevertheless, computationally optimized panels should not be assumed to perform adequately in biological samples solely on the basis of predicted compatibility. Empirical validation remains necessary to assess antibody titration, background fluorescence, spreading-related loss of resolution, and the discrimination of relevant cell populations under the intended staining and acquisition conditions (18, 19).

AI-driven interpretation of high-dimensional immune states. Following spectral unmixing and quality control, computational approaches, including dimensionality reduction, clustering, and machine-learning algorithms, can facilitate the identification and characterization of complex immune populations. Compared with manual gating alone, these methods can help reveal multidimensional phenotypic patterns and continuous cellular states that may be difficult to define using a limited set of predefined markers (122, 123). However, different preprocessing procedures, feature-selection strategies, clustering parameters, and cell-annotation rules can yield different results from the same dataset. Accordingly, computationally identified populations should be interpreted in the context of known immunological biology and, where feasible, validated using independent markers, complementary assays, or orthogonal datasets. Dimensionality reduction and clustering are useful for exploration and hypothesis generation, but they do not by themselves establish the biological identity or functional significance of a cell population.

Predictive modeling and translational applications. Beyond descriptive immune profiling, machine-learning approaches may support the identification of cellular signatures associated with disease activity, treatment response, or clinical outcomes. The cGPS score described in the context of chronic graft-versus-host disease monitoring provides an example of how immune-related measurements can be incorporated into a clinically oriented scoring framework (124). Such examples illustrate the potential of computational analysis to translate complex immune profiles into interpretable measures that may support patient stratification and longitudinal monitoring. However, the predictive performance and clinical utility of a model cannot be inferred from its association with a disease endpoint alone. Model development should account for cohort composition, potential confounding factors, class imbalance, and the risk of overfitting. Independent validation, preferably in external or multicenter cohorts, is needed to establish generalizability beyond the dataset used for model development.

Comparison with previous studies and future priorities. Existing publications illustrate different levels of computational integration, ranging from algorithm-assisted panel optimization and unsupervised immune population discovery to supervised prediction of disease-associated outcomes (123, 125). These approaches address complementary questions and should not be considered interchangeable: panel-design algorithms primarily support experimental planning, unsupervised methods identify patterns within datasets, and supervised models evaluate predefined classification or prediction tasks. Direct comparisons between published models may be limited by differences in instrument configuration, antibody panels, sample processing, cohort characteristics, preprocessing pipelines, and outcome definitions. Future studies should therefore report analytical workflows and model settings transparently, use harmonized benchmarking datasets where possible, and evaluate the robustness of identified populations and predictive signatures across independent cohorts and platforms. Incorporating interpretable models, reproducible computational pipelines, and biologically grounded validation will be essential for translating AI-assisted spectral flow cytometry from exploratory immune profiling into reliable predictive immunology.

### 4.3 Integration with imaging and functional technologies: adding spatial and dynamic dimensions

A major limitation of flow-based analysis of dissociated samples is the loss of spatial context following tissue dissociation. Integration of spectral flow cytometry with imaging-based approaches and functional probes provides opportunities to complement high-dimensional single-cell phenotyping with information on cellular morphology, localization, and functional state.

This complementary strategy is particularly relevant to the study of extracellular vesicles (EVs) and cell–cell communication. Loconte et al. used both imaging flow cytometry and multicolor spectral flow cytometry to characterize tumor-derived EV interactions with immune cells in human PBMCs, demonstrating that different EV-labeling strategies can reveal distinct aspects of EV–cell interactions and uptake (126). Similarly, Gomez et al. demonstrated the utility of imaging flow cytometry for the enumeration and phenotypic characterization of PBMC-derived small EVs in a smoking-related inflammatory disease model (127).

Such multimodal integration may extend spectral flow cytometry beyond static immunophenotyping toward a more comprehensive characterization of cellular communication and immune regulation. When combined with functional or time-resolved assays, these approaches could further link cellular phenotype with localization, functional state, and dynamic cellular behavior.

### 4.4 Integration with single-cell multiomics toward comprehensive immune ecosystem mapping

The integration of spectral flow cytometry with emerging single-cell omics technologies represents an important step toward connecting cellular identity with molecular function. While spectral flow cytometry provides high-throughput measurement of protein expression, immune phenotype, and functional states at the single-cell level, transcriptomic, epigenomic, proteomic, and spatial approaches provide complementary information regarding molecular regulation and tissue organization.

By combining these technologies, researchers can overcome the limitations of individual platforms and achieve a more comprehensive understanding of immune ecosystems across different biological scales. The complementary roles of spectral flow cytometry and emerging technologies are summarized in **Table 3**.

**Table 3:** Integration of spectral flow cytometry with emerging technologies for multidimensional immune profiling.

Integrated technology	Added information	Biological questions addressed	Representative applications
Artificial intelligence	Automated clustering, predictive modeling	Identification of hidden immune states and biomarkers	Immune classification, disease prediction
scRNA-seq	Transcriptomic identity and molecular programs	Linking protein phenotype with gene expression states	Immune cell annotation, disease-associated immune states
Mass cytometry (CyTOF)	Expanded proteomic validation	Cross-platform high-dimensional immune profiling	Disease immune profiling
Spatial profiling	Tissue localization and cellular interactions	Immune–tumor ecosystem organization	Tumor microenvironment mapping
Functional assays	Cellular activity and immune function	Functional validation of immune states	Immunotherapy response, vaccine evaluation

Combined spectral flow cytometry and single-cell RNA sequencing approaches have demonstrated strong correspondence between surface protein expression patterns and transcriptome-defined cellular states, enabling more accurate annotation and functional interpretation of immune populations. Furthermore, integration with transcriptomic and epigenomic profiling has revealed multilayer immune dysregulation in conditions such as vitiligo and post-COVID-19 recovery, insights that cannot be fully captured by individual technologies alone (128–130).

These complementary technologies can therefore be integrated into a multimodal framework in which spectral flow cytometry provides high-dimensional cellular phenotyping, single-cell omics defines underlying molecular programs, and spatial approaches resolve the tissue context of immune states. Such integration enables the relationships among cellular identity, molecular regulation, spatial organization, and functional activity to be examined within a unified analytical framework (**Figure 4**).

**Figure 4:**
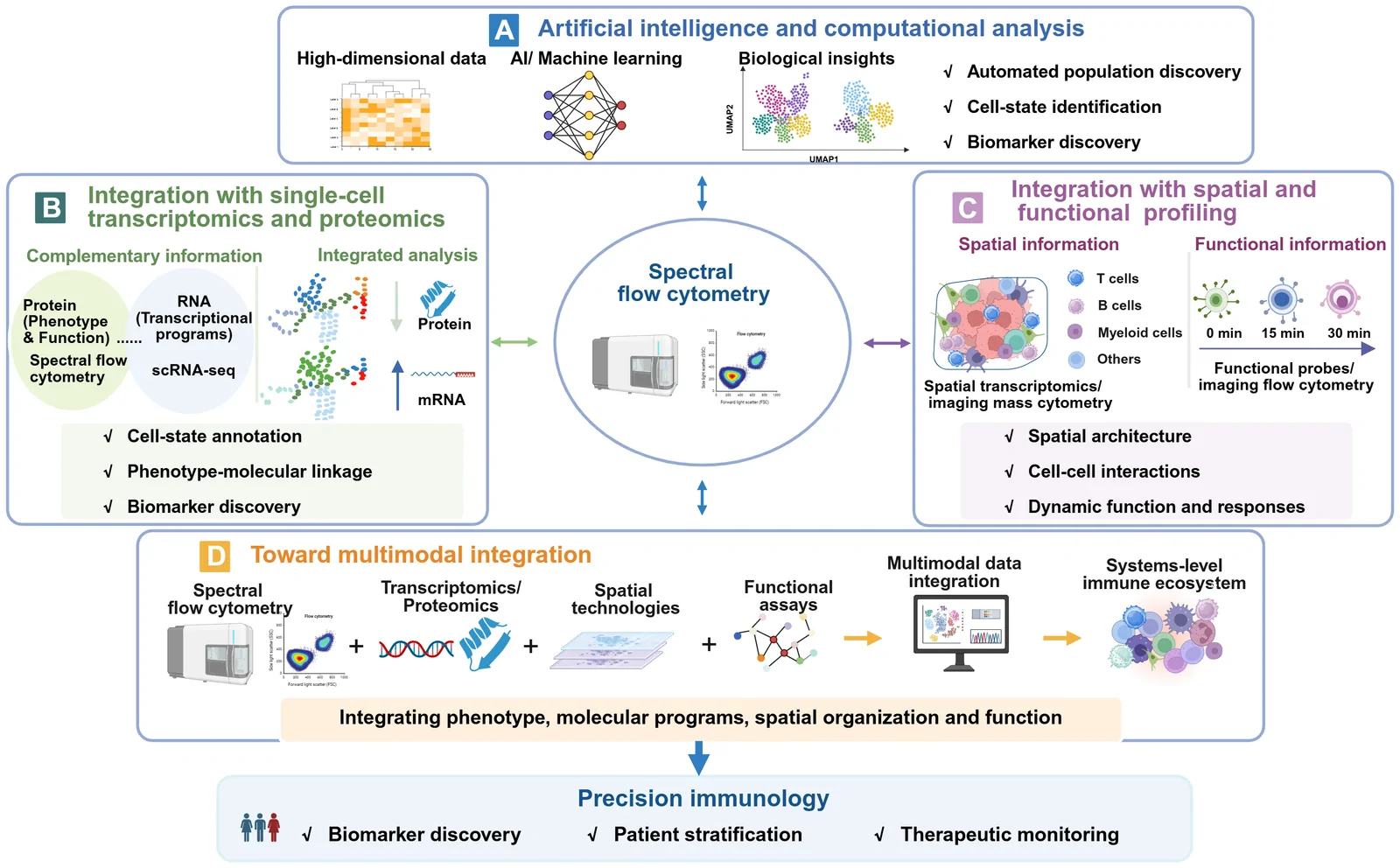
Integration of spectral flow cytometry with emerging technologies toward multimodal immune profiling and precision immunology. **(A)** Artificial intelligence and computational analysis. **(B)** Integration with single-cell transcriptomics and proteomics. **(C)** Integration with spatial and functional profiling. **(D)** Toward multimodal integration for systems-level immune ecosystem characterization, supporting biomarker discovery, patient stratification, and therapeutic monitoring.

Beyond biological discovery, multimodal integration has important translational implications. Platforms combining spectral flow cytometry, sequencing technologies, and spatial proteomic approaches enable comprehensive characterization of complex immune ecosystems, including tumor immune microenvironments, and may support clinical decision-making such as patient selection for adoptive T-cell therapies (131).

### 4.5 Toward multimodal immune profiling platforms

The future trajectory of spectral flow cytometry will likely extend beyond increasing parameter numbers toward integration within multimodal immune profiling ecosystems. By combining high-dimensional phenotyping, computational intelligence, molecular profiling, and spatial information, spectral flow cytometry may provide a comprehensive framework for understanding immune systems across cellular, molecular, and tissue levels.

Such integrated platforms have the potential to redefine precision immunology by enabling improved biomarker discovery, patient stratification, therapeutic monitoring, and personalized treatment strategies. However, successful implementation will require standardized data formats, interoperable analytical pipelines, and rigorous cross-platform validation.

## 5 Current challenges and future perspectives of spectral flow cytometry

### 5.1 Standardization and cross-platform reproducibility

Despite the growing adoption of spectral flow cytometry in immunological research, standardized acquisition and cross-platform reproducibility remain important challenges, particularly in multicenter studies and longitudinal immune monitoring. Unlike conventional flow cytometry, spectral flow cytometry relies on the acquisition of fluorescence emission profiles across multiple detectors and computational spectral unmixing to resolve signals from different fluorophores. Consequently, differences in instrument configuration, detector performance, optical characteristics, fluorophore conjugation, staining conditions, sample preparation, and data-processing procedures can introduce variability into spectral measurements and downstream interpretation (21, 132).

A major source of variability is the generation, validation, and maintenance of reference spectra used for spectral unmixing. Reference spectra may differ depending on the fluorophore conjugate, antibody preparation, staining conditions, sample matrix, and acquisition settings. In particular, tandem dyes may exhibit instability and batch-to-batch variability, highlighting the importance of using appropriate reference spectra for individual conjugates and experimental conditions (31). Moreover, changes in instrument performance over time may affect the consistency of acquired spectral profiles. In longitudinal studies, reference spectra and instrument performance should be monitored over time, because changes in instrument response, fluorophore conjugate properties, or experimental conditions may reduce the suitability of previously acquired reference spectra. When such changes are not identified, spectral mismatches may contribute to residual signals or inaccurate signal allocation during unmixing, potentially introducing systematic variation into longitudinal comparisons. When reference spectra do not adequately represent the fluorescence signatures present in experimental samples, unmixing may become less accurate, potentially resulting in residual signal, distorted marker intensities, or increased uncertainty in the identification of cell populations. These effects may subsequently influence gating, clustering, and comparisons of immune phenotypes across experiments. Thus, reproducibility depends not only on the quality of reference spectra but also on their compatibility with experimental conditions and the stability of instrument performance over time.

Several practical measures may help improve standardization. First, reference spectra should be generated using appropriate single-stained controls and fluorophore conjugates that match those used in the experimental panel, with staining and acquisition conditions kept as consistent as practicable. Second, routine instrument quality control and performance monitoring should be implemented to identify changes that could affect spectral acquisition. Third, harmonized procedures for sample preparation, staining, acquisition, unmixing, and downstream analysis should be established and documented. These measures are consistent with broader flow cytometry recommendations emphasizing instrument standardization, quality control, and appropriate experimental controls (21, 132). Relevant metadata-including instrument configuration, fluorophore and antibody information, reference-control conditions, unmixing settings, and quality-control results-should also be reported to support interpretation and reproducibility. Where feasible, shared reference materials, inter-instrument comparison studies, and common quality-control criteria may further facilitate data harmonization across laboratories.

Importantly, harmonization should not be assumed to make datasets generated on different instruments or under different experimental protocols directly interchangeable. Instead, cross-platform comparisons should account for differences in acquisition and processing conditions and, where possible, include standardized controls or bridging experiments to assess comparability. Establishing transparent reporting practices and validated quality-control frameworks will be essential for improving reproducibility, enabling more reliable multicenter and longitudinal studies, and supporting the broader clinical translation of spectral flow cytometry.

### 5.2 Autofluorescence and complex biological backgrounds

A major challenge for spectral flow cytometry is the accurate characterization and removal of autofluorescence (AF) in complex biological samples. Although spectral acquisition provides additional information for distinguishing overlapping fluorescence signals, cellular autofluorescence originates from endogenous fluorophores and tissue components, including metabolic molecules and structural proteins. Its spectral characteristics and intensity may vary with cell type, metabolic state, tissue composition, sample processing, and disease-associated changes. Consequently, a single AF reference spectrum may not adequately represent the biological heterogeneity of complex samples, particularly those derived from solid tumors, fibrotic tissues, and other highly autofluorescent tissues (31).

AF extraction may become less accurate when the reference spectrum is not representative of the experimental sample or when multiple cell populations exhibit distinct autofluorescence signatures. For example, differences between unstained reference controls and fully stained samples arising from tissue composition, cellular activation, fixation, or other sample-processing conditions may compromise the accuracy of AF modeling. In heterogeneous samples, representing all background fluorescence with a single AF signature may leave residual background signals or cause autofluorescence to be incorrectly attributed to specific fluorophores. These errors can distort measured marker intensities, obscure dim signals, and complicate the identification of rare or phenotypically similar cell populations, ultimately affecting gating, clustering, and downstream biological interpretation (19, 31).

Several practical strategies may help mitigate these limitations. First, unstained controls from the same tissue or cell type should be used to characterize AF, with sample preparation and processing conditions matched as closely as possible to those of the corresponding experimental samples. Where substantial biological heterogeneity or donor-specific variation is expected, AF profiles should be evaluated across relevant samples rather than assuming that a single reference is universally representative. Second, AF extraction and spectral unmixing should be assessed using the fully stained samples, with particular attention to residual background signals, unexpected marker distributions, and the resolution of dim or rare populations. Where a single AF reference is insufficient to represent the sample, the use of multiple AF references or computational approaches that account for heterogeneous AF signatures may improve separation of autofluorescence from fluorophore-derived signals. However, these approaches require appropriate model specification and empirical validation, as unnecessary or poorly characterized AF components may introduce additional uncertainty into spectral unmixing (19, 31).

Finally, improving the reproducibility of AF correction requires transparent reporting of the biological source and processing of samples, the unstained controls used, the AF extraction strategy, and the relevant unmixing and quality-control procedures. Consistent control preparation and documentation are particularly important in longitudinal and multicenter studies, in which differences in tissue composition, sample handling, or acquisition conditions may otherwise be mistaken for biological variation. Further development of representative AF reference libraries, validated multi-component AF models, and standardized quality-control procedures will be important for improving the reliability of spectral flow cytometry in complex tissue-derived samples. For cross-sectional comparisons and meta-analyses of existing datasets, the availability of sufficiently detailed metadata and quality-control information will also be essential for assessing data compatibility and distinguishing biological variation from technical effects.

### 5.3 High-dimensional data analysis and algorithmic challenges

The expansion of spectral parameters provides unprecedented opportunities for immune profiling but also creates substantial analytical challenges. As spectral flow cytometry enables simultaneous measurement of dozens of cellular markers, traditional manual gating strategies become increasingly insufficient for capturing continuous cellular states and complex immune heterogeneity.

Various computational approaches, including dimensionality reduction, clustering algorithms, and machine learning-based annotation, have been increasingly applied to spectral flow datasets. However, differences in preprocessing pipelines, clustering parameters, and annotation strategies may influence biological interpretation and reduce reproducibility between studies.

Therefore, future development requires not only standardized computational workflows but also biologically interpretable analytical frameworks that enable transparent annotation and reproducible interpretation of high-dimensional spectral datasets.

### 5.4 Clinical translation and implementation barriers

Although spectral flow cytometry has demonstrated considerable potential in biomarker discovery and immune monitoring, translation into routine clinical workflows remains challenging. Clinical implementation requires validated analytical performance, including reproducibility, sensitivity, inter-laboratory consistency, and standardized interpretation criteria. Furthermore, while highly multiplexed panels provide comprehensive immune profiling, clinical laboratories often require simplified, robust, and reproducible assays compatible with routine operation.

Future clinical adoption will depend on the development of validated immune monitoring strategies, standardized quality-control procedures, and multicenter validation studies.

### 5.5 Toward integrated immune intelligence platforms

The future development of spectral flow cytometry will likely extend beyond increasing the number of measurable markers toward integrated immune intelligence platforms. By combining spectral profiling with artificial intelligence, single-cell transcriptomics, spatial technologies, and functional assays, spectral flow cytometry may provide a comprehensive framework linking cellular phenotype, molecular regulation, spatial organization, and immune function.

Rather than replacing existing single-cell technologies, spectral flow cytometry will likely serve as a complementary and scalable component of integrated immune analysis ecosystems. Such multimodal approaches may bridge high-dimensional immune profiling with biomarker discovery, therapeutic monitoring, and precision medicine.

## 6 Conclusions: spectral flow cytometry as a foundation for next-generation immune profiling

Spectral flow cytometry represents a fundamental conceptual shift in single-cell analysis, moving beyond conventional fluorescence detection based on discrete optical channels toward computationally resolved spectral profiling. By capturing complete emission spectra and applying spectral unmixing, this technology transforms fluorescence overlap from a technical limitation into an additional dimension of biological information.

Importantly, the major contribution of spectral flow cytometry is not simply the expansion of measurable parameters, but its ability to integrate multiple phenotypic and functional characteristics within individual cells. This capability enables a transition from conventional marker-based classification toward multidimensional characterization of cellular states and immune ecosystems.

Across diverse immunological contexts, spectral flow cytometry has provided new opportunities to dissect immune heterogeneity, including comprehensive immune monitoring, tumor immune ecosystem analysis, autoimmune disease profiling, immune development, vaccination studies, and translational biomarker discovery. By simultaneously resolving lineage identity, activation status, functional programs, and treatment-associated changes, spectral approaches have contributed to a more comprehensive understanding of immune regulation in physiological and pathological conditions.

The future impact of spectral flow cytometry will likely depend increasingly on its integration with complementary technologies rather than on further expansion of marker numbers alone. Combining spectral profiling with artificial intelligence, single-cell transcriptomics, proteomics, spatial technologies, and functional assays may enable the linkage of cellular phenotype with molecular regulation, spatial organization, and functional activity, thereby establishing more comprehensive frameworks for multimodal immune profiling and precision immunology.

Ultimately, spectral flow cytometry should not be viewed as a replacement for conventional flow cytometry or other single-cell technologies, but as a complementary component of an integrated immune profiling ecosystem. By connecting high-dimensional protein-level phenotyping with molecular, spatial, and functional information, spectral flow cytometry has the potential to advance immune analysis from descriptive cellular enumeration toward predictive, mechanistic, and clinically actionable understanding of immune states.
